# Determinants of Practice and Adherence to Infection Prevention and Control of Neonatal Sepsis Among Nurses in Selected Health Facilities of Pwani Region, Tanzania

**DOI:** 10.24248/eahrj.v8i3.814

**Published:** 2025-01-30

**Authors:** Maua Halfani Pendo, Mahamudu Rashid Hussein, Fabiola Vincent Moshi

**Affiliations:** a Department of Clinical Nursing, School of Nursing and Public Health, The University of Dodoma, Tanzania; b Department of Nursing Management and Education, School of Nursing and Public Health, The University of Dodoma, Tanzania.

## Abstract

**Background:**

Although guidelines on neonatal care and infection prevention exist, it is unclear what factors influence nurses’ adherence to infection prevention and control (IPC) in neonatal care. This study aimed to assess the determinants of nurses’ adherence to IPC in neonatal sepsis prevention in the Pwani Region, Tanzania.

**Methods:**

A cross-sectional study with 282 nurses was conducted. Assessing the how Socio demographic characteristics, Health facility factors, attitude of the nurses affect the Nurse’s practices on adherence to IPC for prevention of neonatal sepsis. Data collection methods included a questionnaire and an observation checklist. Bivariate and multivariate logistic regression were employed to determine the factors influencing nurses’ adherence to IPC. Nurse’s adherence to IPC was measured using the mean score whereby those who score above the mean were regarded as adequate adherence. A Probability value of .05 and a 95% confidence interval was regarded as statistically significant.

**Results:**

The present study found that only one-third (37.0%) of the nurses had adequately adhered to IPC. Significant associations were observed between nurse’s adherence to IPC in the prevention of neonatal sepsis and; working experience of 13 to 24, 7 to 12, and 6 months [AOR =5.30, *p<.001*], [AOR=3.9, *p<.024*] and [AOR=3.640, *p<.001*] respectively, >10 years in nursing professional [AOR=2.627, *p<.023*], staffing of 6–10 and 1–5 [AOR=5.992, *p<.001*] and [AOR=3.791, *p<.001*] respectively, 3 and >3 staffs per shift [AOR=3.276, *p<.017*] and [AOR=2.364, *p<.017*) respectively, working at District and regional hospitals [AOR=1.101, *p<.001*] and [AOR= 2.320, *p<.028*] respectively, on-job training [AOR = 2.08, *p<.034*], isolation room availability [AOR=1.783, *p<.042*], SOPs and IPC guidelines availability [AOR=4.320, *p<.004*], sufficient medical equipment and supply [AOR =1.414, *p<.015*] and positive attitude [AOR=1.490, *p<.035*].

**Conclusion:**

The study results indicated Nurse’s adherence to IPC in the prevention of neonatal sepsis is associated with working experience, staffing, healthcare level, on-job training, isolation room availability, current SOPs and IPC guidelines, access to medical equipment/supply, and positive attitude. Strategies should be employed to strengthen the adherence of nurses to IPC guidelines to minimize the morbidity and mortality resulting from neonatal sepsis. Interventional studies from each factor for nurse’s adherence to IPC in the prevention of neonatal sepsis should be of priority.

## BACKGROUND

Neonatal sepsis is a clinical illness that occurs during the first month of life when pathogenic microorganisms (bacteria, viruses, or fungi) are present in typically sterile fluid, such as blood or cerebrospinal fluid (CSF). It is characterized by hemodynamic abnormalities and other systemic clinical symptoms.^1^ Neonatal sepsis remains among the leading cause of neonatal morbidity and mortality worldwide. The rates of neonatal morbidity and mortality resulting from neonatal sepsis is linked to the efficiency and effectiveness of healthcare services.^2^ Infection Prevention and Control (IPC) guideline plays a critical role in managing neonatal sepsis, however, determinants of nurses’ adherence to the guideline are not widely considered in the Pwani region.

Sepsis is a leading cause of death in the first 28 days of life, particularly in sub-Saharan Africa. Neonatal sepsis accounts for 15.0% of all new cases globally, representing 1.3 to 3.9 million new cases of the disease. Additionally, according to the WHO, sepsis kills between 400,000 and 700,000 babies annually.^3,4^ Among hospital-born infants, hospital-acquired infections account for an estimated 4% to 56% of all deaths in the neonatal period, depending on the study and geographical area. An estimated 84% of neonatal deaths due to infections could be prevented through measures such as early diagnosis and timely, appropriate clinical management.^4^

The highest neonatal sepsis incidence rates are in LMICs, particularly in the African region. Severe neonatal sepsis caused 24.0% of neonatal fatalities in Africa, making it the most prevalent condition in LMICs.^5^ In Tanzania, neonatal mortality that is linked to neonatal sepsis stands at 29.0%.^6^ Evidence shows, the mother, neonates, and health services were all linked to neonatal sepsis, which was the primary cause of late neonatal death (29.1%) and the early cause of neonatal death owing to sepsis (21.5%).^7^

Nonetheless, asphyxia at birth, prematurity, low-birth weight, delivery attitudes, mode of delivery, prenatal care, mixed neonatal diets, and cultural practices for umbilical cord care was found to be the major factors contributing to neonatal sepsis worldwide.^2^ Information from the WHO shows that if neonatal sepsis is not identified early and treated quickly, it can lead to septic shock, multiple organ failure, and death.^8^

WHO has taken significant action to reduce neonatal sepsis globally. They include Every Newborn Action Plan program, modernizing healthcare infrastructure, advancing technology, equipping healthcare professionals to identify, treat, and prevent neonatal sepsis, and WHO guidelines for the management of common pediatric diseases.^2,9^

In addition, significant efforts have been made to combat the disease at the community and health facility levels, including increasing community awareness of sepsis, enhancing surveillance, enhancing early detection of sepsis in facilities, ensuring laboratory diagnostics are accessible and affordable, enhancing care quality through sepsis-specific policies, bolstering infection prevention and control during labor, delivery, and postnatal care, improving access to and utilization of vaccines and conducting quality research among newborn populations.^8,9^

Conversely, The Ministry of Health (MoH) in Tanzania is making efforts to address the incidence and prevalence of neonatal sepsis in the country. Various initiatives have been taken such as the adoption of national IPC guidelines, the Reproductive and Child Health Strategy (RCHS) 2005–2010 and Health Sector Strategic Plan IV 2016–2020, National Road Map Strategic Plan to Improve Newborn and Adolescent Reproductive and Maternal Health in Tanzania 2016/One Plan II.^10^

Neonatal sepsis, however, continues to be a substantial concern despite all of these attempts, and both its trend and burden are persistent.^8^ Nurses’ procedures for adhering to the IPC Standard of Practice for the Neonatal Sepsis Prevention and Control Process have received little attention. Furthermore, little is known regarding the ways and degrees to which nurses’ practices influence neonatal sepsis.

In Tanzania, there are limited studies conducted to assess adherence to IPC among nurses working in labour wards and neonatal care units. Many studies have been conducted across different region in Tanzania, however, the focus was to determine the prevalence of neonatal sepsis and associated factors.^7^ Others studies has focus in the general population with no attention to neonates^11^ and some focus in general facilities environment requirement.^12^ According to the current evidence there is no published study that have assessed determinants of nurses’ adherence to IPC and their practices in preventing neonatal sepsis in the Pwani region. Therefore, this study examined the determinants of nurses’ practice and adherence to infection prevention and control of neonatal sepsis in selected health facilities in Pwani region.

## METHODS

### Design

A hospital-based analytical cross-sectional with a quantitative approach was adopted, the design has been adopted since it allows data collection from a large pool of subjects and compare differences between groups, allows observation of many characteristics at once and simultaneously.^13^

### Setting

The study was conducted in the Pwani Region, in Tanzania. Currently, the region has 11 hospitals (10 public and 1 private), 43 health centers (30 public and 13 private), and 319 dispensaries (257 public and 62 private). Among the health services offered in the region are Basic Emergency Obstetric and Neonatal Care (BEMONC) and Comprehensive Emergency Obstetric and Neonatal Care (CEMONC) services. The region also has Neonatal Intensive Care Unit (NICU) and a Pediatric intensive care Unit (PICU). Pwani region has been selected since data regarding neonatal sepsis are limited, it lacked region specific studies that explore the determinants of Nurses adherence to IPC in neonatal sepsis prevention.

### Inclusion Criteria

All registered and enrolled nurses working in labour wards and neonatal units who had working experience of at least six months and above.

### Sample Size

The sample size was determined by using the Cochran formula.^14^ The Proportion used was 43% from the previous study, done by Sayed et al, (2019) in Egypt on 2017.^15^ The calculated sample size was adjusted for non-responses of 10% and the final sample was 414 nurses. However, the calculated sample size was large as compared to the total population of the study participants (nurses). Therefore, the using Cochran formula for sample size calculation in a smaller population, the sample was modified to 282.

### Sampling Procedure

#### Selection of Health Facilities

The region has one Referral hospital (Tumbi) located at Kibaha TC, and each district council has one central district hospital. One Regional hospital (Tumbi) and seven-central district hospital were selected for data collection. Two of district council hospital could not be reached due to administrative issues. In each district council, there is an average of three public health centers,^16,17^ a simple random sampling using a lottery technique was used to select three health centers from each district.

#### Selection of the Participants

Census method was used, whereby, every nurse in the selected unit was recruited into the study making a total sample size of 282 nurses leaving out those involved in pilot study.

### Data Collection Procedures and Tools

Six (6) senior nurses with experience in neonatal care, prior experience in data collection and fluency in the local language were recruited for data collection., They received training for three days on objectives of the study, the methodology, handling ethical issues, and for data collection tools. The training incorporated a role-play to enhance consistency in administering questions and utilizing the observation checklist effectively. The trained research assistants were divided into three groups and each group spent 3 days in each of the selected health facilities collecting data with the checklist for assessing the actual practices for neonatal care through nonparticipatory observation in a ratio of 1:1 (Researcher: participant). Every nurse was observed three times over different shifts (Morning, evening and night shift) in a period of 60 minutes in each observation. The observation was conducted, while, a nurse performing the procedure on neonates, the average score was obtained. However, data collected on the first day were excluded, use of non-participatory method and, and the participants were blinded about the types of procedures that were going to be observed to minimize the Hawthorne effect, whereby, a participants change their behaviours because they are aware they are being observed.^18^

Structured interviews were conducted using a modified questionnaire^19^ that had two parts; first part had 13 items which assessed social demographic characteristics and the second part had 10 items which assessed the nurse’s attitude towards IPC.

The checklist was used to assess health facility factors with a response of Yes and No. The checklist consisted of 8 items which measured availability of medical equipment and supplies, training, sufficient hand washing sink, presence of isolation room, workload, and the availability of SOPs and IPCs guidelines. The items were measured in nominal scale, where by the available items was coded 1 and unavailable item was coded 0.

Nurse’s practices were assessed using 27 items with 6 domains. The first domain was hand washing which had 6 items; handwashing upon entering the NCU/labour ward, before touching a patient, after providing patient care, prior to leaving the NCU/labour ward, and immediately after removing gloves. The second domain was the use of PPE (two items); before providing care and when at risk of being exposed to body fluids. The third domain was dusting and cleanness (five items) which includes resuscitation table cleaned before the next procedure, resuscitation table cleaned after doing the procedure, beds and baby cots cleaned, wards is cleaned using a disinfectant under swipe technique and use of freshly prepared ant septic solution to clean the reusable checkup devices (thermometer, fetoscope, stethoscope, oxygen meter etc.). The fourth domain was cord care (five items); cord is cleaned and dried, fluid or antiseptic applied, cord stump not covered, the cord is checked and record the status and use sterile instruments when performing sterile procedure. The fifth domain was airways suctioning practice (three items); fluid for instillation while airways suctioning, suction catheter/tube changed after single use and suction bottle change. The sixth domain was waste segregation (6 items); sharp box is in place, sharp box used appropriately, proper waste disposal, receiver are in place while performing the procedure, liner bin is in place and liner are used according to colour code. The checklist was developed based on Tanzania Guidelines for the IPC.^20^

### Data Quality Management

Content validity was checked, whereby, the tool was presented to the eight experts in the field and revised based on the expert’s recommendations. Research assistants were trained for data collection to assure they are familiar with the study objectives and tools. Pre-test was done in 10% (28) of the study sample size and those involved were excluded from the actual study.^21,22^ Cronbach’s Alpha test was also used to test for internal consistency (reliability) of the questionnaire and checklist, the score of 0.74 and 0.87 respectively.^23^ On daily basis there were inspection on the completeness, consistency, and accuracy of the collected data.

### Ethical Considerations

Ethical clearance was obtained from the University of Dodoma ethical review board with a Ref No. MA.84/261/02/‘A’/29. Permission to conduct the study was sought from the Pwani region’s Regional Administrative Secretary (RAS) and all District Executive Directors with the Ref. No. Na. DCD.127/265/01 ‘A’/33. Eligible nurses provide Informed consent. Confidentiality, privacy, freedom for giving their responses and the opportunity to withdraw from the study at any time were all upheld. Unique identification numbers were used to identify study participants. Data from the field was secured for approved personnel only.

### Statistical Analysis

Statistical Package for Social Sciences version 25 (SPSS) program was used for data entry and analysis. Outcome variables (practices) and independent variables (socio-demographic characteristics and the health facility factors) data were screened and tested for normality. Parametric measurements were used as all variables were normally distributed. The neonatal sepsis prevention attitude score was calculated based on 10 questions on a five-point Likert scale using descriptive statistics. Chi-square was performed to demonstrate the relationship between the nurse’s practice on adherence to IPC in neonatal care, social demographic data, nurse’s attitude and health facility factors. Binary logistic regression was used to determine the factors associated with nurse’s practice on adherence to IPC in neonatal care. *P* value of <.05 was considered as statistical significance with a confidence interval of 95%.

## RESULTS

### Socio-demographic Information

The average mean age of study participants was 35 years (SD 8.1). More than three-quarters (78.4%) of the participants were female and more than two-thirds (69.1%) of them were living with a partner. Regarding work experience, more than one-third (41.5%) of the participants had more than 2 years of work, while, more than half (56.0%) had a diploma in Nursing (Table 1)

**Table 1:** Nurse’s Socio demographic (N=282)

Variables	Frequency (%)
Age in years	
20-30	94 (33.3)
31-40	119 (42.2)
41-50	51 (18.1)
50-60	18 (6.4)
Gender	
Male	61 (21.6)
Female	221 (78.4)
Marital status	
Living with a partner	195 (69.1)
Not living with a partner	87 (30.9)
Time spent from home to work area	
Less than one hours	187 (66.3)
One hour	57 (20.2)
Greater than one hour	38 (13.5)
Education Level	
Certificate	101 (35.8)
Diploma	158 (56.0)
Advanced Diploma	12 (4.3)
Bachelor and Master	11 (3.9)
Professional category	
Enrolled nurse	99 (35.1)
Registered nurse	183 (64.9)
Years in the nursing profession	
0–3 years	37 (13.1)
4–6 years	125 (44.3)
7-9years	44 (15.6)
10 years and above	76 (27.0)
Working unit	
Labor ward	77 (27.3)
Neonatal ward	51 (18.1)
Neonatal Intensive Care Unit	18 (6.4)
Labor ward and neonatal ward	136 (48.2)
Working experience	
6 months	34 (12.1)
7–12 months	66 (23.4)
13–24 months	65 (23.0)
>24 months	117 (41.5)
Number of total staff in the wards	
1-5	33 (11.7)
6-10	154 (54.6)
>10	95 (33.7)
Number of staff in each shift	
One	58 (20.6)
Two	121 (42.9)
Three	67 (23.8)
More than three	36 (12.8)
Facility level	
Health centers	110 (39.0)
District hospital	150 (53.2)
Regional hospital	22 (7.8)

### Nurses’ Reported Health Facility Factors (N=282)

Standard operation procedures (SOPs) and IPC guidelines were universally available in the visited wards. However, vast majority nurses (95.7%) were overwhelmed with the workload and only 36.5% have received on-job training about neonatal sepsis prevention. One-fifth (20.6%) of nurses reported working in wards with sufficient medical equipment (Table 2).

**Table 2.** Nurses’ Reported Health facility Factors (N=282)

Variables	Yes n (%)	Non (%)
Number of nurses from health facilities with the workload in the wards.	270 (95.7)	12 (4.3)
Number of nurses from health facilities with a shortage of staff in their wards	282 (100)	0 (0.0)
Number of nurses from health facilities who attended on-the-job training (CPD) on neonatal sepsis prevention.	102 (36.5)	179 (63.5)
Number of nurses from health facilities with a large number of patients/overcrowded rooms	143 (50.7)	139 (49.3)
Number of nurses from health facilities with an absence of isolation room for infected neonates	245 (86.9)	37 (13.1)
Number of nurses from health facilities with sufficient hand washing sinks in the unit	198 (70.2)	84 (29.8)
Number of nurses from health facilities with sufficient medical equipment and supply in the wards.	58 (20.6)	224 (79.4)

### Nurses’ Attitude Toward Neonatal Sepsis Prevention

Two-thirds (61.3%) of the nurses strongly believed that immediate action should be taken once newborn is infected in order to prevent further spread of infection. However, half (50.7%) of the participants believed that it is very important to initiate the chain of command if the doctor does not order appropriate and safe interventions for neonates. Also, more than one-third (41.1%) of the participant strongly agreed that it is necessary to comply with IPC standards in neonatal care despite being overwhelmed with activities in the ward, and half (51.4%) of the participants strongly agreed that compliance with IPC standards of care can prevent the spread of microorganisms from patient to patient or from caregiver to patient (Table 3).

**Table 3.** Proportion of Nurses’ Attitude Towards Neonatal Sepsis Prevention in the Pwani Region (N=282)

Variables	Strongly Disagree	Disagree	Neutral	Agree	Strongly Disagree
If I have an infected neonate, I feel this is a high-priority patient demanding immediate action in order to prevent the further spread of infections	0 (0.00)	1 (0.4)	21 (7.4)	87 (30.9)	173 (61.3)
If I receive a newborn baby, I feel comfortable calling or initiating the chain of command if the physician is not ordering appropriate interventions	16 (5.7)	30 (10.6)	67 (23.8)	143 (50.7)	26 (9.2)
I feel it is necessary to adhere to IPC standards in providing care to neonates even though I am over loaded with activities in the ward.	12 (4.3)	25 (8.9)	45 (16)	84 (29.8)	116 (41.1)
I feel referring infected neonates to a high facility is the best way of preventing neonatal sepsis in non-infected neonates	47 (16.7)	55 (19.5)	52 (18.4)	103 (36.5)	25 (8.9)
I feel it is ok to continue providing care from one neonate to another by considering hand washing and other precautions measures.	151 (53.5)	39 (13.8)	39 (13.8)	37 (13.1)	16 (5.7)
I feel it is ok to keep infected neonates with non-infected ones on the same bed if there is scarcity.	165 (58.5)	32 (11.3)	33 (11.7)	37 (13.1)	15 (5.3)
I feel avoidance of artificial nails and removal of rings and wrist watch by health care personnel are important in the prevention of neonatal sepsis.	106 (37.6)	69 (24.5)	39 (13.8)	36 (12.8)	32 (11.3)
I feel adhering to IPC standards of care can help in preventing infections transmission from patient to patient or from nurse to patient	14 (5.0)	28 (9.9)	34 (12.1)	61 (21.6)	145 (51.4)
I feel in my opinion job training and practical guidelines provide sufficient assistance in increasing the knowledge and working competencies of nurses in preventing neonatal sepsis	6 (2.2)	17 (6.0)	45 (16.0)	92 (32.6)	122 (43.3)
I feel it is ok to use the same device which is already used from one patient to another without taking preventive measures when I am overloaded with wards activities	173 (61.3)	29 (10.3)	27 (9.9)	38 (13.5)	15 (5.3)

The mean score of the attitude was 29 (SD=25.3). Using this mean score to dichotomize the attitude variable, two third (66.0%) of the nurses had a negative attitude toward the prevention of neonatal sepsis. Attitude varied with education and years of working experience.

### Nurses’ Practices of Neonatal Sepsis Prevention and Control by Socio-demographic Characteristics

Less than one-third (25.2%) of the participant washed their hands before entering the ward, one-third (33.7%) washed their hands before touching the neonates, and the vast majority (91.1%) of them washed their hands after patient care. More than one-third (35.1%) of the participants used PPE before patient care, and the majority (86.9%) cleaned the resuscitation table/beds before the next procedure. Only 26.6% of participants assessed umbilical cord status, and 36.5% of participants had a receiver when they performed the procedure (Table 4).

**Table 4.** Nurses’ Practices of Preventing and Controlling Neonatal Sepsis (N=282)

Variables	Performed n (%)	Not performed n (%)
Hand washing at entering NCU/labor wards	71 (25.2)	211 (74.8)
Hand washing before touching the patient	95 (33.7)	187 (66.3)
Hand washing after patient care	257 (91.1)	25 (8.9)
Hand washing before leaving the NCU/Labor wards	166 (58.9)	116 (41.1)
Hand washing immediately after removal of gloves	208 (73.8)	74 (26.2)
Use of Personal Protecting Equipment (PPE)when caring	99 (35.1)	183 (64.9)
Use of PPE when exposed to body fluids or cleaning	236 (83.7)	46 (16.3)
Wards are cleaned by using a disinfectant under-wipe technique	255 (90.4)	27 (9.6)
The resuscitation table cleaned before the next procedure	155 (55)	127 (45)
The resuscitation table cleaned after the procedure	232 (82.3)	50 (17.7)
Beds/cots cleaned	245 (86.9)	37 (13.1)
Use of ant-septic solution to clean the reusable check-up devices (thermometer, stethoscope, oxygen meter)	36 (12.8)	246 (87.2
The cord is cleaned and dried	222 (78.7)	60 (21.3)
Fluid or antiseptic applied during cord care	179 (63.5)	103 (36.5)
Cord stump not covered	211 (74.8)	71 (25.2)
The cord is checked and records the status	75 (26.6)	207 (73.4)
Use of sterile instruments when performing a sterile procedure	262 (92.9)	20 (7.1)
Assess skin for changes in color and moisture	239 (84.8)	43 (15.2)
Fluid for instillation is present during airways suctioning	204 (72.3)	78 (27.7)
Suction catheter/tube changed after a single use	231 (81.9)	51 (18.1)
Suction bottle changed/cleaned	202 (71.6)	80 (28.4)
Sharp box is in place	254 (90.1)	28 (9.9)
Sharp box used appropriately	190 (67.4)	92 (32.6)
Proper waste disposal	144 (51.1)	138 (48.9)
The receiver is in place while performing the procedure	103 (36.5)	179 (63.5)
Liner bins are in place	227 (80.5)	55 (19.5)
Liner bins are used according to color code	149 (52.8)	133 (47.2)

The mean practice scores was18 (SD±4.49). Using mean score as cut-off point, approximately two-thirds (62.8%) of the participants had poor practices on adherence to IPC in neonatal care.

### Determinants of Nurses’ Practice of Preventing Neonatal Sepsis

Nurse’s practice of neonatal sepsis prevention and control varied with age, marital status, work experience, attitude, number of staff in the ward and level of health facility (Table 5). Presence of SOPs and IPC guidelines [χ²: 9.26, *P*<.002], in-service training [χ²: 9.44, *P*<.002], availability of sufficient hand wash sinks [χ²: 14.23, *P*=.001] and medical equipment and supplies [χ²: 6.63, *P*=.010] influenced the nurse practices for neonatal sepsis prevention (Table 6). After adjusting for confounders, working experience, years in nursing professional >10 years, number of staff working in the ward or unit, number of shifts associated significantly with nurse’s practices of preventing and control neonatal sepsis (Table 6). A positive attitude [AOR=1.490, *P*<.035], working at regional and district hospital [AOR=1.320, *P*<.028 & AOR=1.901, *P*<.001], on-job training [AOR = 2.08, *P*<.034], and access to medical equipment and supply [AOR =2.414, *P*<.015] influenced nurses’ practice of preventing and control of neonatal sepsis (Table 7).

**Table 5:** Nurses' Practices of Neonatal Sepsis Prevention and Control by Socio-demographic Characteristics (N=282)

Variable	Good practices n (%)	Poor practices n (%)	χ^2^	P-value
Age in years				
20–30	39 (41.5)	55 (58.5)	13.72	.003
31–40	32 (26.9)	87 (73.1)		
41–50	28 (54.9)	23 (45.1)		
>50	5 (27.8)	13 (72.2)		
Gender				
Male	25 (41.0)	36 (59.0)	0.564	.453
Female	79 (35.7)	142 (64.3)		
Marital status				
Living with partner	128 (65.6)	67 (34.4)	17.72	.001
Not living with partner	57 (65.5)	30 (34.5)		
Number of parities				
None	30 (38.5)	48 (61.5)	0.188	.910
One	22 (34.9)	41 (65.1)		
More than one	52 (36.9)	89 (63.1)		
Level of professional education				
Certificate	37 (36.6)	64 (63.9)	5.546	.236
Diploma level	57 (36.1)	101 (63.9)		
Advanced Diploma	3 (25.0)	9 (75.0)		
Bachelor level	5 (55.6)	4 (44.4)		
Master level	2 (100.0)	0 (0.0)		
Professional category				
Enrolled nurse	32 (33.3)	64 (66.7)	5.713	.060
Registered nurse	69 (37.7)	114 (62.3)		
Years in the nursing profession				
Below three years	11 (29.7)	26 (70.3)	5.869	.118
4–6 years	42 (33.6)	83 (66.4)		
7–9 years	23 (52.3)	21 (47.7)		
Above 10 years	28 (36.8)	48 (63.2)		
Type of unit working				
Labor ward	24 (31.2)	53 (68.8)	2.043	.564
Neonatal ward	18 (35.3)	33 (64.7)		
NICU	8 (44.4)	10 (55.6)		
Labor & neonatal wards	54 (39.7)	82 (60.3)		
Working experience				
6 months	17 (50.0)	17 (50.0)	18.66	.001
7–12 months	30 (45.5)	36 (54.5)		
13–24 months	31 (47.7)	34 (52.3)		
>24 months	26 (22.2)	91 (77.8)		
ber of staff in the ward				
1–5	12 (36.4)	21 (63.6)	16.23	.001
6–10	42 (27.3)	112 (72.7)		
More than 10	50 (52.6)	45 (47.4)		
Number of staff per shift				
One	21 (36.2)	37 (63.8)	5.620	.132
Two	51 (42.1)	70 (57.9)		
Three	17 (25.4)	50 (74.6)		
More than 3	15 (41.7)	21 (58.3)		
Facility level				
Health centers	39 (35.5)	71 (64.5)	17.05	.001
District hospital	48 (32.0)	102 (68.0)		
Regional hospital	17 (77.3)	5 (22.7)		
Attitude score				
Positive attitude	49 (51)	47 (49)	9.122	.003
Negative attitude	57 (30.6)	129 (69.4)		

**Table 6:** Nurses' Practice of Neonatal Sepsis Prevention and Control by Health Facility Factors (N=282)

Health facility factors	NURSES' PRACTICES		χ^2^	P-value
	Good practices n (%)	Poor practices n (%)		
Number of nurses from health facilities with current SOPs and IPC guidelines in the wards.				
Yes	104 (39)	163 (61.0)	9.262	.002
No	0 (0.0)	15 (100)		
Number of nurses from health facilities with the workload in the wards.				
Yes	77 (39.1)	120 (60.9)	1.373	.242
No	27 (31.8)	58 (68.2)		
Number of nurses from health facilities with a shortage of staff in the wards.				
Yes	92 (39)	144 (61)	2.754	.971
No	12 (26.1)	34 (73.9)		
Nurses who attended training on neonatal sepsis prevention.				
Yes	78 (43.6)	101 (56.4)	9.44	.002
No	26 (25.2)	77 (74.8)		
Number of nurses from health facilities with a large number of patients in the wards.				
Yes	60 (42)	83 (58.0)	3.21	.073
No	44 (31.7)	95 (68.3)		
Number of nurses from health facilities with the absence of isolation rooms for infected neonates in the wards.				
Yes	60 (33.1)	121 (66.9)	3.02	.082
No	44 (43.6)	57 (56.4)		
Number of nurses from health facilities with sufficient hand washing sink				
No	87 (43.9)	111 (56.1)	14.23	<.001
Yes	17 (20.2)	67 (79.8)		
Number of nurses from health facilities with the sufficiency of medical equipment and supply				
Yes	65 (43.9)	178 (63.1)	6.63	.010
No	39 (29.1)	95 (70.9)		

**Table 7:** Determinants of Nurses' Practice of Neonatal Sepsis Prevention and Control (N=282)

Variables	COR	95% C. I		*P-value*	AOR	95% C.I.		*P-value*
		Lower	Upper			Lower	Upper	
Age in years								
20–30	0.956	0.316	2.896	.937	1.512	0.704	3.524	.411
31–40	1.844	0.608	5.594	.280	1.921	0.848	7.051	.744
41–50	3.165	0.983	10.19	.054	4.981	1.639	13.91	.164
>50	1				1			
Years in Nursing								
Below 3 years	1				1			
4–6 years	1.196	0.539	2.653	.660	1.830	0.839	3.033	.684
7–9 years	2.589	1.031	6.498	.043	3.000	1.538	8.858	1.000
Above 10 years	1.379	0.592	3.210	.456	2.627	1.143	6.038	.023
Working experience								
6 months	3.501	1.573	7.801	.002	3.640	1.702	9.143	.001
7–12 months	3.192	1.662	6.130	.000	3.940	1.892	11.18	.024
13–24 months	2.921	1.521	5.603	.001	5.301	2.301	12.15	<.001
>24 months	1				1			
Number of staff in the unit								
1–5	2.962	1.731	5.072	.000	3.791	1.791	8.032	<.001
6–10	1.523	0.693	3.371	.301	5.992	1.993	18.07	<.001
>10	1				1			
Number of staff per shift								
One	1				1			
Two	1.669	0.775	3.597	.191	1.991	0.861	4.603	.108
Three	2.143	1.110	4.138	.023	2.364	1.164	4.803	.017
>3	2.101	0.888	4.971	.091	3.276	1.232	8.710	.017
Facility level								
Health centers	1				1			
Districts hospital	1.171	0.692	1.963	.561	1.901	1.031	6.382	<.001
Regional hospital	1.234	2.521	20.74	.000	1.320	2.603	25.85	.028
Isolation room								
Yes	1				1			
No	1.557	0.944	2.568	.083	1.783	1.022	3.111	.042
On job training								
Yes	0.443	0.262	0.753	.002	2.083	1.062	4.074	.034
No	1				1			
SPOs and IPC guideline								
Yes	10.30	0.137	1.352	.998	4.321	12.45	14.53	.004
No	1				1			
Sufficient medical equipment and supply								
Yes	1.913	1.166	3.135	.010	2.414	1.201	4.843	.015
No	1				1			
Sufficient hand washing sink								
Yes	3.092	1.691	5.643	.000	4.472	1.813	8.054	.065
No	1				1			
Attitude								
Positive attitude	0.461	0.277	0.765	.003	1.490	1.230	2.044	.035
Negative attitude	1				1			

Compared to nurses who has a working experience of more than 24 months, those working for 6 to 23 months are more likely to have good practice and adherence to IPC for neonatal sepsis [AOR = 3.640, *P*<.001]. Compared to nurses who worked in nursing for less than 3 years, those working for more than 10 years in wards are more likely to have good practice and adherence to IPC for neonatal sepsis [AOR = 2.627, *P*<.023]. Compared to nurses who worked in wards with more than 10 staff, those working in wards with less staff were more likely to have good practice and adherence to IPC for neonatal sepsis [AOR=3.791, *P*<.001]. Compared to nurses who worked in wards with one staff per shift, those who working in ward with three or more staff are more likely to have good practice and adherence to IPC for neonatal sepsis [AOR=3.791, *P*<.001]. Nurses who work in district or regional hospital are more likely to have good practice and adherence to IPC for neonatal sepsis [AOR=1.901, *P*<.001, AOR=2.603, *P*<.028] compared to those in health centre. Nurses who have on-job training are more likely to have good practice and adherence to IPC for neonatal sepsis [AOR=2.083, *P*<.034]. Nurses who work in ward with SOPs and IPC guides and sufficient medical equipment are more likely to have good practice and adherence to IPC for neonatal sepsis [AOR=4.321, *P*<.004, AOR=4.472, *P*<.065]. Nurses who have positive attitude are more likely to have good practice and adherence to IPC for neonatal sepsis [AOR=2.083, *P*<.034].

## DISCUSSION

The study aimed to examine the determinants of nurses’ practice and adherence to IPC in neonatal sepsis prevention in the Pwani region. Nurses’ practice in the wards is a fundamental factor in reducing complications that can occur in sick newborns. In this case, nurses are required to demonstrate their skills in various aspects of neonatal sepsis prevention and control by adhering to the IPC standards for better neonatal health.

The current study revealed that nurses in the surveyed health facilities have poor adherence to IPC guidelines and exhibit limited practice of neonatal sepsis prevention. The results are in line with other studies conducted elsewhere.^15,24,25^ However, Desta et al., (2018)^26^ reported that less than half of the nurses surveyed had unacceptable infection prevention practices in the labor ward. This difference may be due to different study participants and access to resources in hospitals. Poor neonatal sepsis prevention practice call for redesigning the interventional program to change the nurses’ practices. Failure to address the identified gap may lead to increased risk of healthcare-associated infections, morbidity, mortality, emerging of antimicrobial resistance (AMR) and financial burdens for both healthcare system and families.

Attainment of high education level and long working experience in the field of nurse were key factors for nurses’ good practice in preventing neonatal sepsis. Nurses who possess higher level of education are more likely to provide evidence-based care, make sound clinical decisions, and deliver high-quality patient care.^27^ These results are similar to the study conducted by Desta et al., (2018)^26^ who found that the practice of sepsis prevention increase as the level of education increase. However, in the aspect of years of working in nursing professionals the study differs from the current finding.^26^ The difference in practices based on the number of years of working in nursing could be due to other factors such as on-job training. Therefore, in order to decrease risks of acquiring neonatal sepsis and minimizing complication from sepsis nurses should be trained to higher level qualification, subjected to different in-services training to acquire the experience on managing sepsis.

Concerning working experience and years in the profession: current study found that as the number of years worked in nursing increased, so did the standard professional practices among nurses. This is expected as the new staff look over the repeated activities from the senior nurses it improves their clinical decision-making abilities, adherence to evidence-based guidelines, and communication skills.^28,29^ This result is supported by studies conducted by other researchers.^15,30,31,32^

Availability of adequate number of staff in the wards and an increased number of nurses per shift (three and more) influenced nurse practices for the prevention of neonatal sepsis. Sufficient number of staff provides a greater opportunity to share some of the workload.^33^ This result is consistent with a study by Nyikuri^34^ who found that structural organizational factors such as adequate staffing facilitated nurses’ adherence to infection control in the neonatal unit.

Nurses working in the hospitals had good practices than those working at health centers. This could be due to the fact majority of the nurses with high education level were working at the hospital level. At hospital level there is an increase in a number of specialized nurses and other professionals.^35^ The findings are consistent with the study by Michael, et al.^36^ Neonates being attended at the lower-level health facilities are at higher risk of acquiring sepsis, therefore, the need to strengthen the care services.

On-job training equip nurses with necessary skills and knowledge needed to perform their duty effectively. In line with other studies,^25,26,32^ in-service training had a positive impact on nurses’ practice of neonatal sepsis prevention.

The availability of medical equipment, supplies, SOPs and IPCs guidelines is crucial in improving the efficiency, and quality of the services. The findings of this study support what have been reported by the other study,^25,26^ that the lack of resources for IPC was a major factor in the poor practice of IPC in healthcare settings.

Positive attitude promotes patient-centered care, create a positive working environment that foster collaboration and teamwork, this, in turn, lead to good nursing practices in prevention of neonatal sepsis. The positive association between practice and attitude towards prevention of neonatal sepsis reported in this study is in line with the findings of Rizany et al., (2018).^37^

### Study Strengths and Limitations

Current study is describing the determinants of nurses’ practices on adherence to IPC in neonatal sepsis prevention using a suitable statistical model. It shows the regional data hence, it can be used as a comparative data based with another region or national database. However, it’s a cross-sectional study, it cannot identify the cause-effect relationship, whereby, the connection between two events where one event (the cause) directly leads to another event (the effect).^38^ Also, the study was based in one region and used non-probability sampling, therefore, generalization of the finding should be cautious.

### Practice and Research Implications

The current study provides locally generated evidence by highlighting the importance of health facility factors and nurses’ education, experience, on-job training and positive attitude in improving nurses’ practices for prevention of neonatal sepsis. It also provides the base for further interventional research programs that address factors impacting nurse’s adherence on IPC guideline in neonatal sepsis prevention. It also paves a way for other regions to establish their own study to compare the existing differences, that may impact the resources distribution in the region.

## CONCLUSION

Despite availability of SOPs and IPC guidelines, poor practice in prevention of neonatal sepsis persist among nurses. Key determinants of good practice included working experience, staffing levels, on-the-job training, availability of medical equipment and supply, and positive attitude towards prevention of neonatal sepsis. There is a need to develop strategies to strengthen nurses’ adherence to SOPs and IPC guidelines, and transform their attitude regarding prevention of neonatal sepsis.
